# Icaritin Inhibits Collagen Degradation-Related Factors and Facilitates Collagen Accumulation in Atherosclerotic Lesions: A Potential Action for Plaque Stabilization

**DOI:** 10.3390/ijms17020169

**Published:** 2016-01-28

**Authors:** Zong-Kang Zhang, Jie Li, De-Xin Yan, Wing-Nang Leung, Bao-Ting Zhang

**Affiliations:** 1 School of Chinese Medicine, The Chinese University of Hong Kong, Hong Kong 999077, China; maxzhangzk@cuhk.edu.hk (Z.-K.Z.); lijie@cuhk.edu.hk (J.L.); 2 Shanghai Clinical Center of Cardiovascular and Cerebrovascular Diseases in Traditional Chinese Medicine, Shanghai Tenth People’s Hospital, Tongji University, Shanghai 200072, China; rszbt@hotmail.com

**Keywords:** Icaritin, atherosclerosis, plaque stabilization, matrix metalloproteinase, collagen

## Abstract

Most acute coronary syndromes result from rupture of vulnerable atherosclerotic plaques. The collagen content of plaques may critically affect plaque stability. This study tested whether Icaritin (ICT), an intestinal metabolite of Epimedium-derived flavonoids, could alter the collagen synthesis/degradation balance in atherosclerotic lesions. Rabbits were fed with an atherogenic diet for four months. Oral administration of ICT (10 mg·kg^−1^·day^−1^) was started after two months of an atherogenic diet and lasted for two months. The collagen degradation-related parameters, including macrophages accumulation, content and activity of interstitial collagenase-1 (MMP-1), and the collagen synthesis-related parameters, including amount and distribution of smooth muscle cells (SMC) and collagen mRNA/protein levels, were evaluated in the aorta. ICT reduced plasma lipid levels, inhibited macrophage accumulation, lowered MMP-1 mRNA and protein expression, and suppressed proteolytic activity of pro-MMP-1 and MMP-1 in the aorta. ICT changed the distribution of the SMCs towards the fibrous cap of lesions without increasing the amount of SMCs. Higher collagen protein content in lesions and aorta homogenates was observed with ICT treatment compared with the atherogenic diet only, without altered collagen mRNA level. These results suggest that ICT could inhibit the collagen degradation-related factors and facilitate collagen accumulation in atherosclerotic lesions, indicating a new potential of ICT in atherosclerotic plaques.

## 1. Introduction

Disruption of atherosclerotic plaques triggers thrombus formation and consequent acute coronary syndromes including unstable angina, acute myocardial infarction, and sudden death [1]. It is well established that a large lipid-rich core underlying a thin fibrous cap containing few smooth muscle cells (SMCs) and collagen content predispose the plaques to rupture [2,3]. Pathological and angiographic studies have determined that the ruptured thin fibrous cap of a coronary atheroma causes acute fatal thrombus formation [4,5]. The SMCs secrete extracellular matrix molecules, including collagen, elastin, and proteoglycans, to form the fibrous cap [1]. Interstitial forms of collagen providing biomechanical strength to the fibrous cap usually protects thrombogenic material in the plaque’s core from contact with coagulation factors in blood [6]. Depletion of fibrillar collagens from the fibrous cap leads to cap thinning and a predisposition to rupture. A family of matrix metalloproteinases (MMPs) derived from inflammatory cells (macrophages, foam cells) in atherosclerotic plaques is predominantly responsible for the depletion of matrix components [7,8,9]. However, this matrix reduction may also be attributed to suppressed collagen synthesis due to a decrease in SMCs proliferation and/or an inhibition of their synthetic function [2,3]. Imbalance between collagen synthesis and degradation is proposed to be a key determinant of plaque disruption, *i.e.*, the trigger of most acute coronary events [1,10]. Studies in rabbits and non-human primates have shown a promising potential of statins to stabilize plaques by suppressing macrophage accumulation, reducing MMP expression, increasing SMCs number and collagen protein content in atheromatous plaques [10,11]. Clinical trials have also established an inverse association between statin therapy and cardiovascular events and mortality [12,13]. However, statin treatment fails to prevent approximately 50%–70% of coronary events, even with prolonged or aggressive therapy [6]. Some adverse effects, such as myopathy and increase in hepatic transaminase increase, may compromise patients’ compliance with long-term statin therapy [14,15]. Therefore, it requires developing alternative approaches that may stabilize atherosclerotic plaques in safety. 

Flavonoids are the most common type of polyphenolic compounds found in varity fruites and vegetables. Population studies have shown that dietary flavonoids are associated with lower cardiovascular disease mortality [16,17]. Flavonoid-rich Epimedium L. (Berberidaceae) has been traditionally used as a medicinal herb to treat cardiovascular diseases, musculoskeletal diseases, and gonad dysfunctions in Asia for thousands of years [18,19]. Our clinical trials found no obvious side effects of oral Epimedium-derived Flavonoids (EFs) on major systems for two years in postmenopausal women [20]. EFs have been shown to inhibit the macrophage activity and foam cell formation [21], and suppress the expression and/or activity of MMPs in non-vascular cells [22,23]. EFs have also been reported to promote collagen synthesis in osteoblast [24] and chondrocytes [25]. This implies that EFs may alter the parameters related to collagen synthesis and degradation in atherosclerotic plaques in a manner to facilitate the plaque stablity. However, EFs contain a series of flavnoid compounds with a parent core. The clinical compliance with EFs would be low due to the high oral dosage. It is necessary to identify an active molecular compound which might exert beneficial effects for stablizing atherosclerotic plaques.

**Figure 1:**
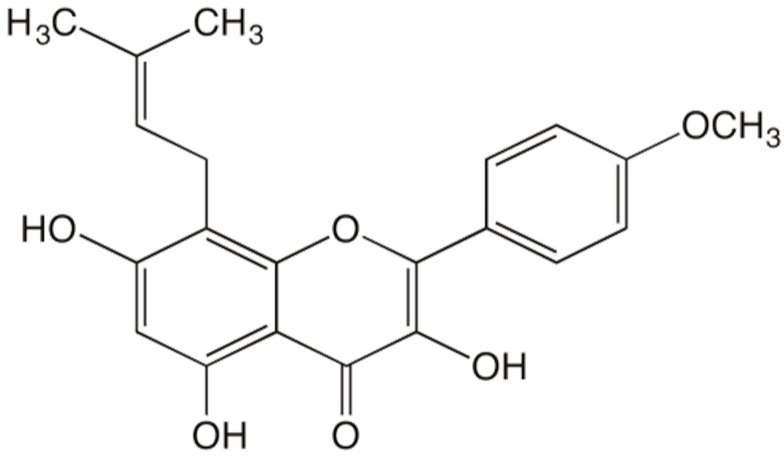
The structural formula of icaritin.

Icaritin (ICT), an identified intestinal metabolite of EFs, has demonstrated many biological activities in vascular disorders (Figure 1) [26,27,28]. ICT has been shown to inhibit the activation of mouse peritoneal macrophages [29], suppress the expression of MMPs in human osteosarcoma cells [30] and increase the mRNA level of collagen type I in human bone mesenchymal stem cells [31]. However, the efficacy of ICT on parameters related to collagen synthesis and degradation in atherosclerotic plaque and underlying mechanisms are not known.

This study tested the hypothesis that ICT could inhibit collagen degradation-related parameters, including macrophages accumulation, content and activity of interstitial collagenase-1 (MMP-1), and improve collagen synthesis-related parameters, including the amount and distribution of the SMCs and collagen mRNA level, in rabbit atheromatous lesions.

## 2. Results

### 2.1. ICT Reduced Plasma Lipid Levels

New Zealand White rabbits were randomly assigned to the control, high cholesterol diet (HC), or high cholesterol diet plus ICT administration (HC+ICT) group. The animals of the control group consumed standard rabbit chow. The animals of the HC and HC+ICT groups consumed a high cholesterol diet for two months to induce atherosclerotic lesion formation. Peripheral blood was collected to measure the concentrations of plasma total cholesterol (TC), triglyceride (TG) and low-density lipoprotein cholesterol (LDL-C). At the beginning of the experiment, there was no significant difference in plasma TC (control: 2.74 ± 0.17, HC: 2.71 ± 0.28, HC+ICT: 2.68 ± 0.27 mmol/L), TG (control: 1.03 ± 0.15, HC: 0.94 ± 0.08, HC+ICT: 1.07 ± 0.10 mmol/L) and LDL-C (control: 1.23 ± 0.13, HC: 1.26 ± 0.18, HC+ICT: 1.23 ± 0.15 mmol/L) levels among the three groups. After two months of an atherogenic diet, plasma TC (HC: 5.91 ± 0.44, HC+ICT: 6.36 ± 0.37 mmol/L), TG (HC: 2.18 ± 0.05, HC+ICT: 1.92 ± 0.27 mmol/L) and LDL-C (HC: 2.81 ± 0.06, HC+ICT: 3.00 ± 0.21 mmol/L) levels were significantly elevated compared with the control group (TC: 2.27 ± 0.22, TG: 0.71 ± 0.08, LDL-C: 1.16 ± 0.14; *p* < 0.05 for all 3 markers). Subsequent oral administration of the ICT (10 mg·kg^−1^·day^−1^) for two months apparently decreased plasma TC, TG and LDL-C levels compared with the HC group (*p* < 0.05 for all three markers) (Table 1). 

**Table 1:** Plasma lipids at the termination of experiments.

	TC (mmol/L)	TG (mmol/L)	LDL-C (mmol/L)
Control	2.92 ± 0.27	0.73 ± 0.05	1.32 ± 0.12
HC	5.43 ± 0.04 *	1.81 ± 0.14 *	2.71 ± 0.08 *
HC+ICT	4.83 ± 0.13 ^#^	1.51 ± 0.06 ^#^	2.19 ± 0.10 ^#^

* *p* < 0.05, between control and HC groups; ^#^ *p* < 0.05, between HC and HC+ICT groups. *n* = 9.

### 2.2. ICT Inhibited Macrophages Accumulation and MMP-1 Protein Expression, and Up-Regulated Collagen Protein Content in Atherosclerotic Lesions

**Figure 2:**
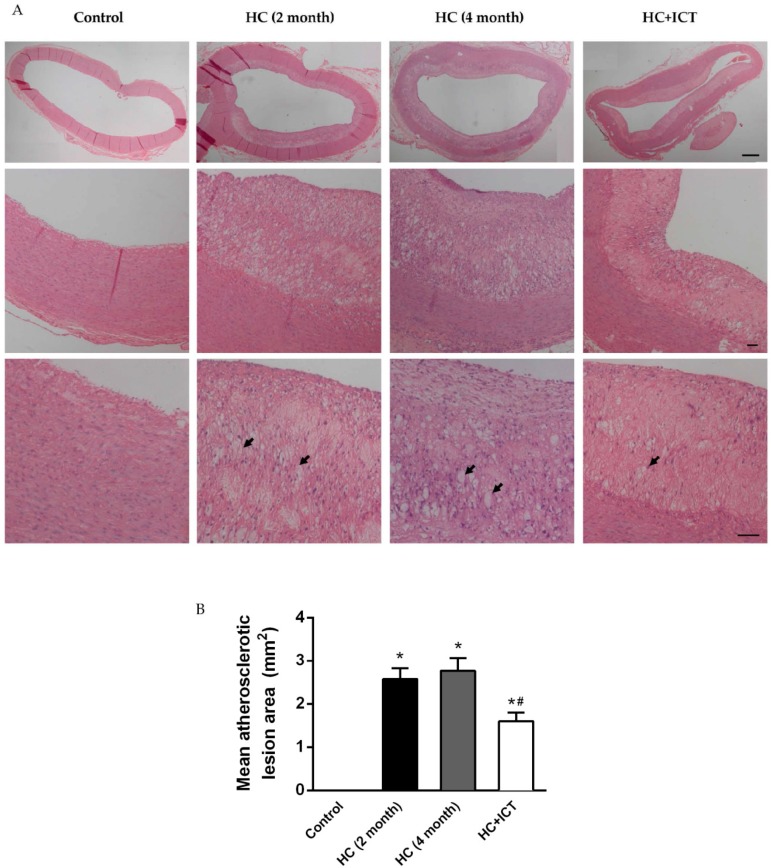
High cholesterol (HC) diet induced atherosclerotic lesion formation in rabbit aorta. (**A**) Representative cross sections of aorta stained by hematoxylin and eosin (H&E). Thickened intima of aorta and fatty streaks presented in all HC-fed groups compared with that from rabbits consuming standard chow (Control). Black arrow: foam cell. Scale bar = 200 µm (**top** panel), 50 µm (**middle** and **bottom** panels); (**B**) Quantitative analysis of atherosclerotic lesion area. * *p* < 0.05, between control and HC groups; ^#^ *p* < 0.05, between HC (four month) and HC+ICT groups. *n* = 2 in HC (2 month) group, *n* = 9 in the other three groups. Data were means ± SEM.

**Figure 3:**
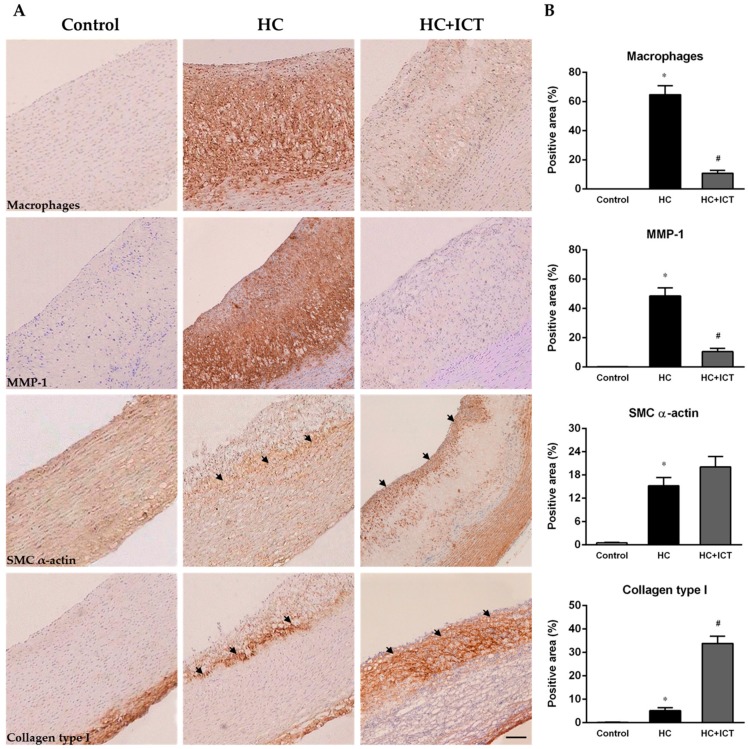
ICT inhibited macrophage accumulation and MMP-1 protein expression, and up-regulated collagen protein content in intima of aorta. (**A**) Representative images of immunohistochemistry for macrophages CD68, MMP-1, SMC α-actin and Collagen type I in cross sections of aorta from control, HC, and HC+ICT groups. Scale bar = 200 µm. Black arrow: smooth muscle cells (third panel) and collagen type I (fourth panel); (**B**) Quantitative analysis of intimal macrophages-, MMP-1-, SMC α-actin- and Collagen type I-positive area reported as percentage of the entire intima area. * *p* < 0.05, between control and HC groups; ^#^ *p* < 0.05, between HC and HC + ICT groups. *n* = 9. Data were means ± SEM.

Atherosclerotic lesion formation in the aorta was evaluated after two months and four months of an atherogenic diet ingestion. The intima of the aorta was severely thickened. Fatty streaks, composed of macrophage-derived foam cells intermingled with smooth muscle cells and extracellular matrix, were observed in all HC-fed groups. There were no obvious atherosclerotic lesions in control group, and was no significant change in the atherosclerotic lesion area between two months and four months on the HC diet. While the lesion area in the HC+ICT group was significantly lower than that in the HC group (Figure 2). More detailed analysis of atherosclerotic lesion composition was performed by immunohistology for macrophages, MMP-1, SMC α-actin, and collagen distribution and expression in the neointima. The group fed with atherogenic diet had numerous macrophages accumulation from the base to the shoulder region of lesions. MMP-1 localized predominantly in the macrophages region of lesions. The percentages of intimal area positive for macrophages (64.7% ± 6.05%, *p* < 0.05) and MMP-1 (48.5% ± 5.61%, *p* < 0.05) were significantly higher in the HC group compared with the control group (macrophages: 0.05% ± 0.01%, MMP-1: 0.2% ± 0.05%). Treatment with ICT largely inhibited macrophage accumulation (10.8% ± 2.06%, *p* < 0.05) and MMP-1 protein expression (10.5% ± 2.32%, *p* < 0.05) in the lesions compared with the untreated group (Figure 3). 

Following an atherogenic diet ingestion, SMCs localized mostly in the boundaries between neointima and media suggesting vulnerable plaques. Distribution of collagen type I had a same pattern as SMCs in the HC group. The percentages of intimal area positive for SMC α-actin (15.2% ± 2.14%, *p* < 0.05) and collagen type I (5.18% ± 1.23%, *p* < 0.05) were significantly higher in the HC group compared with the control group (SMC α-actin: 0.5% ± 0.13%, collagen type I: 0.2% ± 0.05%). Though ICT administration did not significantly influence the level of SMC α-actin (20.1% ± 2.68%, *p* > 0.05) compared with the HC group, it changed the distribution of SMCs by forming a dense layer of SMC accumulation in the cap region of lesions, suggesting relatively stable plaques. Treatment with ICT largely up-regulated collagen type I protein content (33.8% ± 3.16%, *p* < 0.05) in the lesions (Figure 3).

### 2.3. ICT Lowered MMP-1 mRNA Level and Unaltered Collagen mRNA Level in Neointima

**Figure 4:**
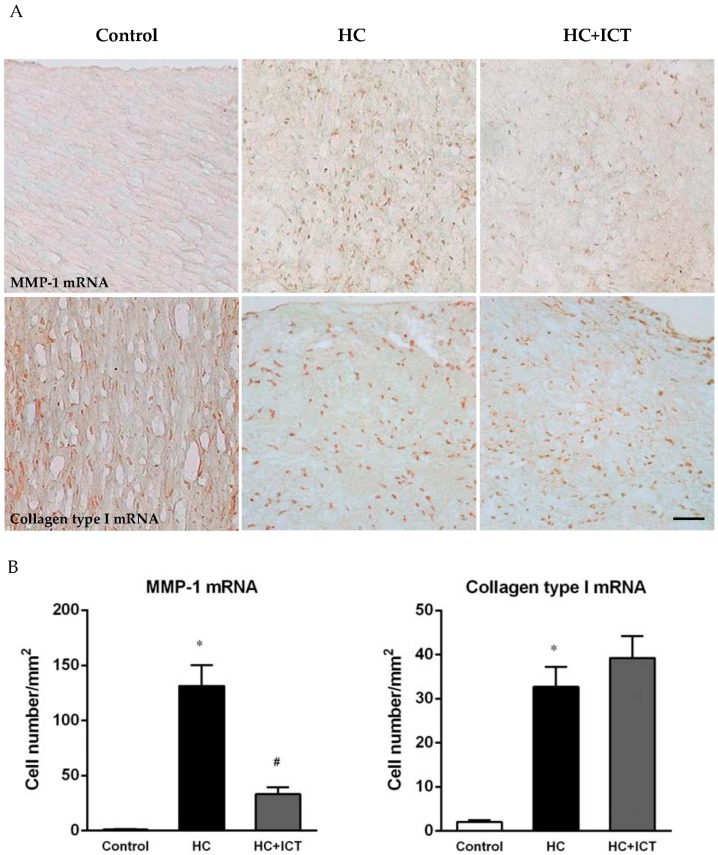
ICT lowered MMP-1 mRNA expression in intima of aorta. (**A**) Representative images of *in situ* hybridization for MMP-1 and collagen type I mRNA in cross sections of aorta from control, HC, and HC+ICT groups. Scale bar = 50 µm; (**B**) Number of MMP-1 mRNA- and collagen type I mRNA-positive cells per mm^2^ intima. * *p* < 0.05, between control and HC groups; ^#^ *p* < 0.05, between HC and HC+ICT groups. *n* = 9. Data were means ± SEM.

*In situ* hybridization showed more cells positive for MMP-1 mRNA (131 ± 18.9 cells/mm^2^, *p* < 0.05) in neointima following an atherogenic diet than the controlled aortic sections, for which MMP-1 mRNA expression was nearly undetectable (1.0 ± 0.36 cells/mm^2^). ICT administration apparently lowered MMP-1 mRNA-positive cells in neointima (33.1 ± 6.18 cells/mm^2^, *p* < 0.05). Cells positive for collagen type I mRNA were predominantly in adventitia and media in the controlled aortic sections and significantly increased in neointima following an atherogenic diet (32.7 ± 4.54 cells/mm^2^, *p* < 0.05 compared with the control: 2.02 ± 0.41 cells/mm^2^). Treatment with ICT had no significant difference in collagen type I mRNA-positive cells compared with the HC group (Figure 4).

### 2.4. ICT Lowered MMP-1 mRNA Levels with Collagen mRNA Levels Unchanged in Aorta Homogenates

**Figure 5:**
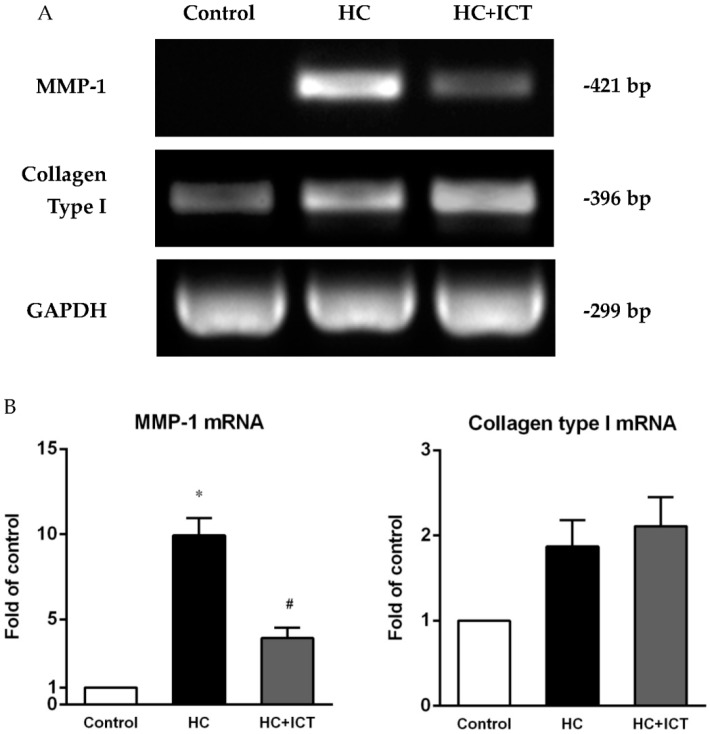
ICT lowered MMP-1 mRNA expression in aorta homogenates. (**A**) Representative bands of RT-PCR for MMP-1 and collagen type I. GAPDH as internal control; (**B**) Expression of MMP-1 and collagen type I mRNA was normalized to GAPDH intensity and presented as the fold of the control group. * *p* < 0.05, between control and HC groups; ^#^ *p* < 0.05, between HC and HC+ICT groups. *n* = 9. Data were means ± SEM.

Consistent with the results from *in situ* hybridization, RT-PCR detected significantly higher MMP-1 mRNA expression in aorta homogenates following an atherogenic diet than that in the control group. This elevation was apparently attenuated by the treatment with ICT (*p* < 0.05). There was no statistical difference in collagen type I mRNA level in aorta homogenates between the ICT-treated and untreated groups (Figure 5).

### 2.5. ICT Suppressed MMP-1 Protein Level and the Proteolytic Activity of Pro-MMP-1 and MMP-1, and Up-Regulated Collagen Protein Content in Aorta Homogenates

**Figure 6:**
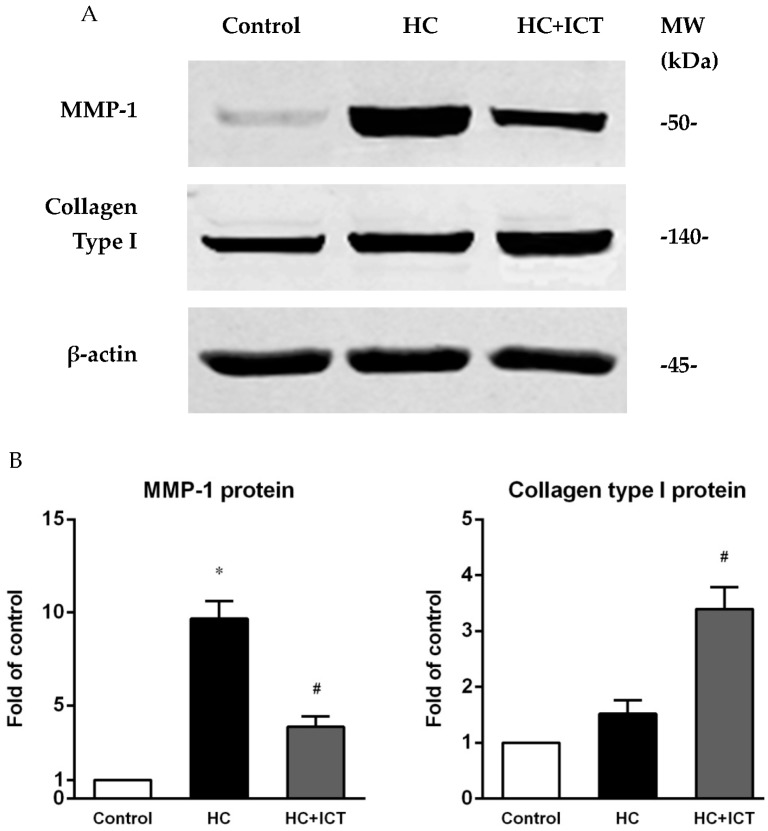
ICT suppressed MMP-1 and up-regulated collagen type I protein contents in aorta homogenates. (**A**) Representative bands of western blot for MMP-1 and collagen type I. β-actin as internal control. MW: molecular weight; (**B**) Expression of MMP-1 and collagen type I protein was normalized to β-actin intensity and presented as the fold of the control group. * *p* < 0.05, between control and HC groups; ^#^ *p* < 0.05, between HC and HC+ICT groups. *n* = 9. Data were means ± SEM.

MMP-1 protein level (detected by western blot) was largely elevated in aorta extracts following atherogenic diet compared with the control group (*p* < 0.05). Treatment with ICT had lower MMP-1 protein level than that in the untreated group (*p* < 0.05). Feeding with an atherogenic diet also elevated collagen type I protein level with no statistical difference when compared with the control group. ICT administration had significantly higher protein level of collagen type I than that in the HC group (*p* < 0.05) (Figure 6).

**Figure 7:**
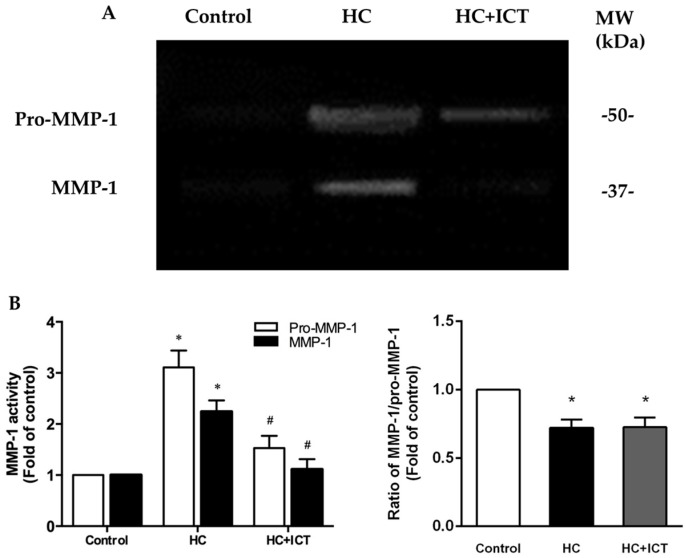
ICT suppressed the proteolytic activity of MMP-1 in aorta homogenates. (**A**) Representative images of gelatin zymography for pro-MMP-1 and MMP-1 activity. Cleared areas indicated proteolysis of the gelatin. MW: molecular weight; (**B**) Intensity of cleared areas corresponding to pro-MMP-1 and MMP-1, as well as the ratio of MMP-1/pro-MMP-1, were normalized to the control group and reported as the fold of the control group. * *p* < 0.05, between control and HC groups; ^#^ *p* < 0.05, between HC and HC + ICT groups. *n* = 5. Data were means ± SEM.

We next observed the proteolytic activity of MMP-1 by gelatin zymography. For the aorta extracts, the lysis bands at 49 and 37 kDa coincide with pro-MMP-1 and MMP-1 (Figure 7A) [32], and agree with reported mobility and lysis activity of pro-MMP-1 and MMP-1 [33]. The gelatinolytic activity of both pro-MMP-1 and MMP-1 increased following an atherogenic diet. This elevation was significantly inhibited by ICT treatment. However, ICT did not influence the ratio of MMP-1/pro-MMP-1 (Figure 7B).

## 3. Discussion

The cholesterol fed rabbits have been widely used as a hypercholesterolemic model to study the effects of drugs on atherogenesis despite the distribution patterns of atherosclerosis being different in human diseases [34]. Collagen content contributes critically to the atherosclerotic plaques stability. The present study demonstrated an increased accumulation of interstitial collagen in the atherosclerotic lesions of ICT-treated animals. Several underlying mechanisms may account for this observation.

### 3.1. ICT Inhibits Collagen Degradation-Related Factors, including Macrophage Accumulation, MMP-1 Expression and the Proteolytic Activity of Pro-MMP-1 and MMP-1

Macrophages play an important role in the pathogenesis of acute coronary syndromes [35]. Lesional macrophages overexpress various proteinases, including members of the MMP family such as interstitial collagenases (MMP-1, MMP-8, and MMP-13), gelatinases (MMP-2 and MMP-9), and stromelysin (MMP-3) [36,37,38], which potently cleave triple helical fibrillar collagen. Thus, macrophages and MMP-related proteolysis within the atherosclerotic plaques may undermine the fibrous cap and accelerate plaque rupture [6]. The present study demonstrates that ICT could inhibit collagen degradation-related molecular and cellular events, evidenced by the inhibited macrophage accumulation, lowered MMP-1 mRNA and protein expressions, and suppressed proteolytic activity of pro-MMP-1 and MMP-1 in the aorta of the ICT group. The decreased MMP-1 activity might result from the inhibited pro-MMP-1 activity by ICT, accounting for an unaltered MMP-1/pro-MMP-1. These inhibitory effects of ICT on the features of macrophages and MMP-1 may either result from a direct action on the macrophages or suppression of MMP production [1].

### 3.2. ICT Does Not Promote Collagen Synthesis, but Changes Collagen Distribution to the Fibrous Cap

A thin fibrous cap containing few SMCs and collagen content always exists in the ruptured human atherosclerotic plaques [2,3]. The present study showed that most of SMCs and collagen localized at the base of the lesions, instead of the fibrous cap following a high-cholesterol diet. This could render the plaque less resistant to rupture. ICT could not promote collagen synthesis, evidenced by the unaltered SMCs and collagen mRNA levels, whereas ICT could facilitate a dense accumulation of SMCs and collagen in the fibrous cap area.

### 3.3. The Underlying Mechanism for the Promoted Collagen Accumulation in the Atherosclerotic Lesions by ICT

Collagen content which critically contributes to atherosclerotic plaque stability, is determined by a dynamic balance between its synthesis and degradation. The present study demonstrated no altered collagen mRNA level but higher collagen protein content in the ICT group. This inconsistency could be explained by the inhibited collagen degradation-related factors and changed collagen distribution, though the collagen synthesis was not altered.

### 3.4. Cholesterol Lowering Effects Could Not Fully Explain the Influence of ICT on Plaque Biology

In experimental models, systemic hypercholesterolemia is associated with severe monocytosis [39], which consequently gives rise to macrophages in atheromata [40]. In the present study, ICT slightly reduced plasma TC, TG and LDL-C by 11%, 16% and 19%, respectively. Previous study has shown similar results of EFs, the underlying mechanisms of which could be explained by the inhibitory effects of EFs on the inflammatory response and the p38 MARK signaling pathway [41]. While there were large reductions in macrophage accumulation (83%), and MMP-1 protein expression (78%), and an increase in collagen protein content (six-fold) in the ICT-treated lesions, these could not be simply explained as the secondary effect to lower LDL-C levels. Moreover, among the rabbits that have similar lipid profiles, the lesion areas of macrophages, MMP-1 and collagen type I are two- to three-fold different. These results do not support that ICT improves atherosclerotic lesion biology dependent on effects on plasma lipids. Interestingly, cholesterol lowering-independent effects of plaque stabilization were observed with simvastatin treatment [42].

### 3.5. Uniqueness of ICT Action in Comparison with Current Therapeutics

Nowadays, statins are the primary drug of choice for treating cardiovascular disease. Cholesterol lowering therapy with statins has proven its efficiency in atherosclerosis treatment. Statins also have been shown to suppress SMCs migration and proliferation [43]. Moreover, it has been reported that statins could dominantly promote collagen synthesis-related molecular and cellular events, evidenced by the increased collagen level and unaltered macrophage and MMP-9 content in statin-treated patients [44]. In contrast, ICT could remarkably inhibit collagen degradation-related molecular and cellular events, and change SMCs and collagen distribution, showing the uniqueness of ICT action in atherosclerotic lesions.

### 3.6. Limitations

In the present study, we investigated the effects of ICT on plaque biology of a single dose, which proved optimal in the prevention of steroid-induced osteonecrosis in our previous study [28]. We aimed to understand whether ICT would simultaneously have a cardioprotective action at the dose that also produces the largest protection of bone tissue. Of course, the optimal dose for plaque stabilization still needs to be confirmed in a extended study. In addition, the current study systematically observed changes in MMP-1 (collagenase-1) at transcriptional, post-translational, and proteolytic levels. Other members of the MMP family, such as MMP-8 (collagenase-2), MMP-13 (collagenase-3), MMP-3 (stromelysin-1), and MMP-9 (gelatinase-B), should be investigated subsequently. Dual immunohistochemistry and *in situ* hybridization should be performed to allow the delineation of which cell types (macrophages, smooth muscle cells) express MMP-1, and relevant associations with collagen expression. The effect of ICT on monocytes/macrophage invasion, proliferation, apoptosis, and foam cell formation should be investigated systemically. Furthermore, endothelial dysfunction is a key trait of atherosclerosis [45]. Preserving endothelial function is important to attenuate atherosclerosis process [46]. The effect of ICT on proliferation of endothelial cells and endothelial function should be evaluated.

## 4. Materials and Methods

### 4.1. Animal Experiments

Thirty male New Zealand White rabbits (2.0–2.2 kg) were housed separately. All animal experiments were conducted according to the protocol approved by the Animal Ethics Committee of Tongji University (2002J007B). Rabbits were randomly assigned either to the control, or high cholesterol diet (HC), or high cholesterol diet plus ICT (U-sea Biotech, Shanghai, China) administration (HC+ICT) group (*n* = 10 each). The animals of the control group consumed standard rabbit chow. The animals of the HC and HC+ICT groups consumed a high cholesterol diet (1% cholesterol, 5% lard, 10% yolk powder and 84% standard rabbit chow) for 2 months to induce atherosclerotic plaque formation. One rabbit from the control, HC and HC+ICT groups, respectively, were euthanized for atherosclerotic lesion evaluation. Remaining animals of the HC and HC+ICT groups (*n* = 9 each) continued to consume a high cholesterol diet (0.5% cholesterol, 2.5% lard, 5% yolk powder and 92% standard rabbit chow) for further 2 months. Simultaneously, ICT (10 mg·kg^−1^·day^−1^) was orally administered to the rabbits of the HC+ICT group for 2 months.

### 4.2. Plasma Lipids Levels

Peripheral blood sample was collected under anesthesia by intramuscular injection of ketamine (35 mg/kg)/xylazine (7 mg/kg). Plasma total cholesterol (TC), triglyceride (TG) and low-density lipoprotein cholesterol (LDL-C) concentrations were measured by enzymatic assays (Sigma-Aldrich, St. Louis, MO, USA).

### 4.3. Tissue Preparation

Rabbits were euthanized by injecting sodium pentobarbital (120 mg/kg) intravenously. Meanwhile, heparin (100 U/kg) was injected to prevent blood coagulation. The portion (20 mm) between the ascending aorta and the aortic arch was excised and rinsed briefly with sterile saline solution. One segment (4 mm) was fixed with 4% paraformaldehyde and embedded in paraffin by conventional procedures for H&E staining (atherosclerotic lesion area) and immunohistochemistry. Another adjacent portion (4 mm) was snap-frozen with OCT compound in pre-chilled isopentane bath cooled with liquid nitrogen for fresh-frozen sections for *in situ* hybridization. Remaining portion of aorta (12 mm) was stored in −80 °C for reverse transcription-polymerase chain reaction (RT-PCR), western blot and zymography analysis. 

### 4.4. Immunohistochemistry

Paraffin-embedded cross sections (4 µm) were deparaffinized in xylene and rehydrated in a graded ethanol series. The antigen retrieval was done by incubating sections in boiling 10 mM citrate buffer (PH 6.0) for 30 min, followed by cooling at room temperature for another 20 min. Endogenous peroxidase activity was inactivated by incubating the sections for 10 min in 0.3% (*v*/*v*) hydrogen peroxide/methanol. Sections were then incubated with 1% (*w*/*v*) bovine serum albumin (BSA) in phosphate-buffered saline (PBS) for 30 min for blockage of non-specific protein binding. Incubation with mouse monoclonal antibodies against human macrophages CD68 (ZhongShan Biotechnology, Beijing, China), MMP-1 (Antibody Diagnostica Inc., Greenwich, CT, USA), α-smooth muscle actin (Maxim Bitotech, Rockville, MD, USA), and collagen type I (ZhongShan Biotechnology, Beijing, China) was applied for 3 h at room temperature. Then, sections were incubated with biotinylated anti-mouse Immunoglobulins (DAKO A/S, Glostrup, Denmark) for 30 min and followed by incubation with horseradish peroxidase-labeled streptavidin solution (Vector Laboratories, Burlingame, CA, USA) for 30 min. Sections were rinsed in PBS after each incubation step. The reaction was visualized with diaminobenzidine DAB (DAKO A/S, Glostrup, Denmark) as the substrate. Sections were counterstained with hematoxylin and mounted. Negative control staining was performed by omitting primary antibody.

### 4.5. In Situ Hybridization

Detection of MMP-1 and collagen type I mRNA on the aortic cross sections was performed with the *in situ* hybridization kit following manufacture’s protocol (Boster, Wuhan, China). Three oligonucleotide probes corresponding to rabbit mRNA for MMP-1 were synthesized: 5′-AGTCTGGAAATACCTGGAAAACTACTACAA-3′, 5′-TGAAGAAGCCCAGGTGTGGGGTGCCTGATG-3′, 5′-CTAACCTTTGATGCTATAACTACATTTAGG-3′. Three oligonucleotide probes corresponding to rabbit mRNA for collagen type I were synthesized: 5′-GGCCACCGCCCTCCTGACGCACGGCCAAGA-3′, 5′-ACTTCAGCTTCCTGCCCCAGCCACCTCAAG-3′, 5′-TGATGCCAACGTGGTTCGTCACCGTGACCT-3′. All oligonucleotide probes were labeled with digoxin (Boster, Wuhan, China).

Frozen sections (10 µm) were fixed with 4% paraformaldehyde, washed in PBS and air dried. Sections were incubated with 1% (*v*/*v*) hydrogen peroxide/methanol for 30 min to inhibit endogenous peroxidase activity. After washes in RNase-free water, sections were treated with pepsin for 2 min, followed by washes in PBS and RNase-free water. Sections were then incubated for 3 h at 39 °C with pre-hybridization buffer (Boster, Wuhan, China) and for overnight at 39 °C with 1 ng/µL of the oligonucleotides in hybridization buffer (Boster, Wuhan, China) in a humid chamber. After incubation, sections were washed with a graded series of saline sodium citrate (SSC) solution (1 × SSC: 150 mM sodium chloride, 15 mM tri-Sodium citrate dihydrate, pH 7.0) and incubated with blocking solution for 30 min at 37 °C. Sections were then incubated with biotinylated mouse antibody against digoxin for 60 min at 37 °C. After washes in PBS, sections were incubated for 20 min at 37 °C with SABC-POD system and biotinylated peroxidase, respectively. Peroxidase activity was revealed by DAB (DAKO A/S, Glostrup, Denmark). Sections were dehydrated and mounted. Negative control staining was performed by omitting oligonucleotide probes.

### 4.6. Reverse Transcription-Polymerase Chain Reaction

Total RNA was isolated from rabbit aortas with TRIzol reagents (Invitrogen, Carlsbad, CA, USA). Four microgram of total RNA was reversely transcripted with Oligo-dT and Maloney murine leukemia virus (M-MLV) reverse transcriptase (Sangon, Shanghai, China). Four microliters of reverse transcription (RT) product was amplified with Taq DNA polymerase (Sangon, Shanghai, China) using a primer pair specific either to MMP-1 (forward primer: 5′-CAGGGAGATCATCGTGACAA-3′, reverse primer: 5′-CCGCATGTAGAACCTGTCTT-3′) [47], or to collagen type I (sense primer: 5′-CTCAGACCCAAGGACTATGA-3′, antisense primer: 5′-CAGACGCATGAAGGCAAGTT-3′) [48]. A primer pair of rabbit glyceraldehyde-3-phosphate dehydrogenase (GAPDH) was used as control (sense primer: 5′-GTGAAGGTCGGAGTCAACG-3′, antisense primer: 5′-GGTGAAGACGCCAGTGGACTC-3′) [49]. The final reaction mixture (20 µL) was initially heat-denatured at 94 °C for 5 min followed by polymerase chain reaction (PCR) amplification using the following conditions: denaturation for 1 min at 94 °C, annealing for 1 min at 55 °C, and elongation for 1 min at 72 °C for 30 cycles for MMP-1 and 25 cycles for collagen type I and GAPDH. This procedure was followed by a final extension at 72 °C for 5 min. PCR product was 421 base pairs for MMP-1, 396 base pairs for collagen type I, and 299 base pairs for GAPDH. Each final PCR product sample (10 µL) was electrophoresed on a 1.2% agarose gel and visualized by ethidium bromide. The relative intensities of the bands were quantified by a densitometric analysis software (Tanon GIS 3.71, Shanghai, China) and expressed as the fold of the control value.

### 4.7. Western Blot

The aortic tissue was homogenized in Rapid Immunoprecipitation Assay (RIPA) buffer (20 mM Tris-HCl (pH 7.5), 150 mM NaCl, 1 mM Na_2_EDTA, 1 mM EGTA, 1% NP-40, 1% sodium deoxycholate, 2.5 mM sodium pyrophosphate, 1 mM β–glycerophosphate, 1 mM Na_3_VO_4_, and 1 µg/mL leupeptin) (Cell Signaling Technology, Danvers, MA, USA), mini-complete protease inhibitor cocktail (Roche Diagnostics GmbH, Mannheim, Germany), 4 mM dithiothreitol (DTT), and 1 mM phenylmethylsulfonyl fluoride (PMSF). Homogenates were clarified by centrifugation at 13,000× *g* for 30 min at 4 °C. Protein concentrations were determined using the Bradford protein assay (Bio-Rad Laboratories, Hercules, CA, USA). Then, protein (40 µg) of each sample was separated by 10% SDS polyacrylamide gel electrophoresis (SDS-PAGE) and transferred onto a nitrocellulose membrane with a Bio-Rad’s Transblot at 200 mA for 2 h. The membrane was blocked with 5% non-fat dry milk in Tris-buffered saline/0.1% Tween-20 (TBST) overnight at 4 °C and incubated with mouse monoclonal antibodies (all from Santa Cruz Biotechnology, Santa Cruz, CA, USA) against MMP-1 (1:200), collagen type I (1:200), or β-actin (1:2000) for 2 h at room temperature. After washing with TBST, the membrane was incubated with IRDye 800 CW goat anti-mouse IgG (LI-COR, Lincoln, NE, USA) for 1 h at room temperature. The membrane was scanned by Odyssey (LI-COR, Lincoln, NE, USA) and the intensities of the bands were quantified by Odyssey v3.0 (LI-COR, Lincoln, NE, USA). The protein levels were normalized by β-actin expression and presented as the fold of the control value. 

### 4.8. SDS-PAGE Zymography

Protease activity of MMP-1 was detected by zymographic analysis. Samples were homogenized in the RIPA buffer without PMSF. Equal amounts of protein (20 µg) were prepared in nondenaturing sample buffer and loaded into the well of the SDS-PAGE gel containing 1 mg/mL gelatin (Sigma-Aldrich, St. Louis, MO, USA). Gelatin is not only degraded by gelatinases (MMP-2 and -9), but to a lesser extent by collagenase (MMP-1) [32,50,51]. After electrophoresis, gels were washed 2 × 15 min in 2.5% Triton X-100 solution to remove SDS. Then, gels were incubated with zymographic development buffer (50 mM Tris, pH 7.4, 10 mM CaCl_2_, and 0.05% Brij 35) overnight at 37 °C and stained with Coomassie blue solution (Sigma-Aldrich, St. Louis, MO, USA). Cleared areas indicated proteolysis of the gelatin. Zymograms were quantified by densitometry with the Gel Doc 1000 system (Bio-Rad Laboratories, Hercules, CA, USA) and presented as the fold of the control value.

### 4.9. Quantitative Analysis for Histology

Brightfield images were captured using a Nikon Eclipse 80i fluorescence microscope (Nikon Corporation, Tokyo, Japan) equipped with a color digital video SPOT Flex camera (15.2, 64 Mp, Shifting Pixel; Diagnostic Instruments Inc., Sterling Heights, MI, USA). The whole cross section of aortas was imaged under same microscope settings to ensure identical background levels. The images were evaluated using digital image analysis program (ImageJ 1.49, National Institutes of Health, Bethesda, MD, USA). The atherosclerotic lesion area in H&E staining sections (mm^2^) was measured. Staining was considered positive if the staining intensity exceeded the specific threshold that was defined by negative control staining. For immunohistology, CD68-, MMP-1-, α-smooth muscle actin-, and collagen type I-positive areas within intima were quantified as a percentage of the entire intima area (%). Analysis of *in situ* hybridization for MMP-1 and collagen type I mRNA (cell number/mm^2^ intima) was performed manually by 2 different observers who counted all positive cells within intima in a blinded manner. The intraclass correlation coefficient (ICC) between the two observers was 0.96 suggesting excellent reliability.

### 4.10. Statistics

Analysis of multiple groups was performed using a one-way ANOVA with the Bonferroni correction for multiple comparisons. Values are expressed as means ± S.E.M. Differences were considered significant at *p* < 0.05.

## 5. Conclusions

This study demonstrates that ICT could inhibit the collagen degradation-related factors and facilitate collagen accumulation in atherosclerotic lesions initiated by hyperlipidemic diet in rabbits, indicating a new potential of ICT in atherosclerotic plaques. The underlying mechanisms might be related to the suppression of macrophage accumulation and collagenase (MMP-1) expression and activity. Higher collagen content might render plaques more resistant to rupture.
